# Catalysing (organo-)catalysis: Trends in the application of machine learning to enantioselective organocatalysis

**DOI:** 10.3762/bjoc.20.196

**Published:** 2024-09-10

**Authors:** Stefan P Schmid, Leon Schlosser, Frank Glorius, Kjell Jorner

**Affiliations:** 1 Institute of Chemical and Bioengineering, Department of Chemistry and Applied Biosciences, ETH Zurich, Zurich CH-8093, Switzerland; 2 Organisch-Chemisches Institut, Universität Münster, 48149 Münster, Germany; 3 National Centre of Competence in Research (NCCR) Catalysis, ETH Zurich, Zurich CH-8093, Switzerland

**Keywords:** catalyst design, machine learning, modelling, organocatalysis, selectivity prediction

## Abstract

Organocatalysis has established itself as a third pillar of homogeneous catalysis, besides transition metal catalysis and biocatalysis, as its use for enantioselective reactions has gathered significant interest over the last decades. Concurrent to this development, machine learning (ML) has been increasingly applied in the chemical domain to efficiently uncover hidden patterns in data and accelerate scientific discovery. While the uptake of ML in organocatalysis has been comparably slow, the last two decades have showed an increased interest from the community. This review gives an overview of the work in the field of ML in organocatalysis. The review starts by giving a short primer on ML for experimental chemists, before discussing its application for predicting the selectivity of organocatalytic transformations. Subsequently, we review ML employed for privileged catalysts, before focusing on its application for catalyst and reaction design. Concluding, we give our view on current challenges and future directions for this field, drawing inspiration from the application of ML to other scientific domains.

## Introduction

Since the beginning of the 21st century, organocatalysts [1] have established themselves as a third group of homogeneous catalysts, next to biocatalysts [2] (enzymes) and transition metal-based catalysts [3]. In particular, enantioselective organocatalysis has shown an impressive rise in the last decades, owing to the tunability of catalysts and different modes of activation, enabling a manifold of different transformations [4–5]. The development of the field, driven by many researchers, led to the award of the Nobel Prize to List and MacMillan in 2021 ‘for the development of asymmetric organocatalysis’. Organocatalytic transformations have also seen the transition to industrial processes for the production of a variety of pesticides and medicinal compounds, as recently reviewed [6–9].

Despite the prominence of organocatalytic reactions, catalyst development has so far mostly been conducted guided by intuition of skilled organic chemists. Given that organocatalytic reactions are governed by different competing interactions, the influence of a change in molecular structure is often non-trivial, even for highly experienced experts. Thus, intuition-guided catalyst development is regarded as suboptimally efficient and furthermore highly subjective to the experience of the chemists carrying out the study [10–15]. Considering the demand of organocatalysts, their accelerated and reliable development is highly desirable [16]. In the spirit of accelerated discovery, the development of organocatalysts has been augmented with computational catalyst design [17–18]. Multiple programs for automated catalyst simulation have been developed in the last decade. Notable examples include the development of ACE (Asymmetric Catalyst Evaluation) [19–20], AARON (Automated Reaction Optimiser for New Catalysts) [21] or CatVS (Catalyst Virtual Screening) [22]. Such tools have been extensively reviewed in the past years [23–25]. Based on a known mechanism, the tools calculate the energies of relevant species either via force field or quantum chemical methods to assess the properties of a reaction such as activation energies or selectivity. Irrespective of the degree of automation, in silico calculations are often less time-sensitive than wet-lab experiments and can be used to reduce the number of required experiments. As such, these methods contribute to the acceleration of catalyst discovery, for example through high-throughput virtual screening.

Predating these computational techniques is the desire to understand and explain experimental outcomes in organic chemistry with physicochemical descriptors. A prominent early example are Hammett parameters, developed in 1937 [26–27], that relate substituent parameters to the equilibrium constant of the deprotonation of a substituted benzoic acid. The derived substituent parameters are used to gain insight into the mechanism of reactions by observing the influence of substituents on a reaction outcome. However, Hammett parameters have shown to not fully describe observed trends. Therefore, complementary representations capturing other properties of a molecule have been derived (vide infra) [28].

While traditional linear free energy relationships such as those using Hammett parameters used linear models, the emergence of ML has led to the development of more complex algorithms, better suited for extracting hidden patterns in data. The ability of ML to efficiently capture complex relationships allows to extract influences on catalyst properties and thus makes it suited towards the accelerated design of chemicals and materials, including organocatalysts [29]. Due to this potential, an increasing number of research groups have used ML to predict and develop new organocatalytic reactions.

This review aims to provide a critical overview of developments in ML specifically for organocatalysis over the last decade, with a focus on its applications. We aim to provide a starting point to catalysis researchers who are interested in ML as well as an assessment of critical challenges to more experienced ML users. We will first give a primer on ML, equipping experimentalists with the knowledge necessary to follow the developments in the field. The rest of the review is divided into three parts: (1) ML for reactivity and selectivity prediction, (2) ML for the design of privileged organocatalysts and (3) ML for catalyst and reaction design. Ultimately, the review will give an outlook on the authors’ expectation of the future of the field.

## Review

### 1. Primer on ML

#### 1.1 Data

**Figure 1:**
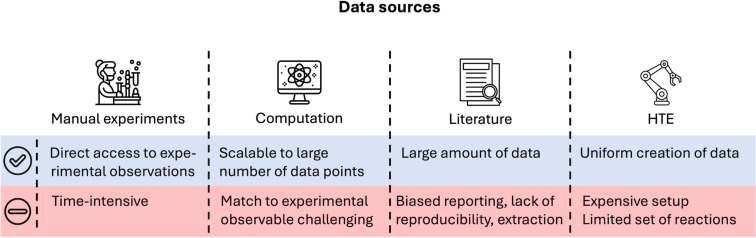
Schematic depiction of available data sources for predictive modelling, each with its advantages and disadvantages. Icon ‘Manual experiments’ made by Eucalyp from flaticon.com. This content is not subject to CC BY 4.0. Icon ‘Computation’ made by Wichai.wi from flaticon.com. This content is not subject to CC BY 4.0. Icon ‘Literature’ made by Muhammad Atif from flaticon.com. This content is not subject to CC BY 4.0. Icon ‘HTE’ made by Nuricon from flaticon.com. This content is not subject to CC BY 4.0. Icon ‘Pros’ made by Aldo Cervantes from flaticon.com. This content is not subject to CC BY 4.0. Icon ‘Cons’ made by Yogi Aprelliyanto from flaticon.com. This content is not subject to CC BY 4.0.

The foundation for any predictive model is the underlying data. It represents the source from which the model extracts relevant patterns and relations. Therefore, the size and quality of the underlying dataset will determine the model’s predictive capabilities. To obtain high predictive accuracy for a broad range of problems, a data set is sought which covers the problem space comprehensively. This does not only encompass the chemical diversity of the included molecules, but also the range of results, e.g., reactions with low, medium and high selectivity [30]. Predictions for data points outside of the applicability domain, e.g., the region which is not sufficiently covered by the provided training data, are less reliable, which is why an appropriate choice of training data is paramount for predictive modelling. Depending on the problem at hand, different sources of data are available (Figure 1).

Apart from experimental data, the creation of large amounts of in silico data is possible with sufficient computational resources [31–32]. While this approach is useful in cases where the experimental determination is challenging, some experimental properties, like the reaction yield, remain elusive to be reliably computed due to the myriad of factors (side-reactions, impurities, solvation effects, interface effects,...) that influence this observable [33–34]. Another pitfall regarding computational data is its accuracy with respect to the ground truth, in particular for multiple factors relevant throughout catalysis, such as non-covalent interactions (NCIs) for organocatalysis or spin properties for transition metal catalysis [35–36]. While most quantities can in principle be computed with the highest accuracy using advanced tools, the associated computational cost needs to be considered [18,24].

Therefore, the use of experimental data is advantageous as less assumptions have to be made and the quantity of interest is directly represented. The results of a great number of experiments can be found in literature, as well as patents. Manual curation of this data is possible, but for larger amounts of data it is usually impractical. Therefore, automated extraction tools have been reported yielding the data in a structured format suitable for ML [37–41]. While some important efforts have been made to establish uniform data reporting standards [42–43], they are getting picked up by the community rather slowly. With data from experiments conducted by different scientists under varying conditions and adhering to various standards, reproducibility remains a major challenge in organic chemistry and restricts the applicability of literature data for statistical modelling [30]. Despite emerging high-throughput experimentation (HTE) pipelines [44–45], large datasets of high-quality are still scarce. While multiple large datasets are available for transition metal catalysis [46–48] and biocatalysis [49–51], they are however not common for organocatalysis. Therefore, much research has been devoted to develop models that perform well on the available small data sets [52–53].

#### 1.2 Representation

In order to be processed by any ML model, the data needs to be provided in a machine-readable way. Unlike chemists who typically use drawings of Lewis structures to represent molecules, computers require a numerical representation of the molecular structure. Since the information that describes the input directly influences what relationships a model can learn from the presented data, different representations might be suitable depending on the task.

**Figure 2:**
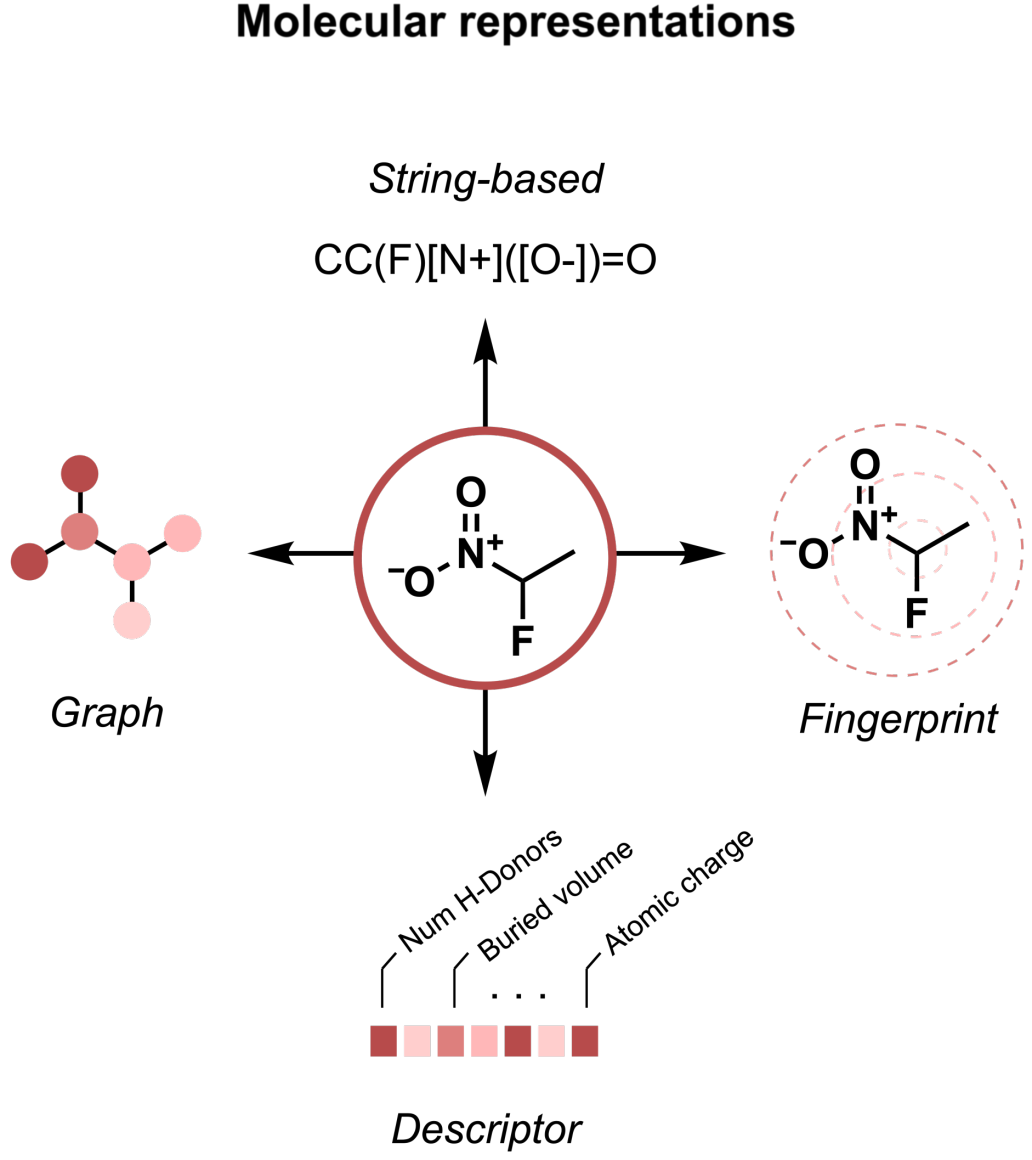
Schematic depiction of different kinds of molecular representations for fluoronitroethane. Among the most common representations are string-based notations, such as SMILES, structural fingerprints, like the ECFP, or molecular graphs. Another way of encoding a molecule is through descriptors that often contain steric or electronic properties.

Besides the most commonly used string-based representations, such as the Simplified Molecular Input Line Entry Specification (SMILES) [54] and fingerprints like the extended connectivity fingerprint (ECFP) [55], molecules can be directly represented as graphs (Figure 2).

In graphs, the atoms and bonds are represented as nodes, and edges, respectively [56]. While these kind of representations are well suited for the description of most organocatalysts with distinct bonds, they have limitations when describing coordination compounds as commonly found in transition metal catalysis for example [57].

Another kind of representation that has found considerable application for ML in organocatalysis, is the use of descriptors. These are sets of numerical or categorical values to encode a molecule. A plethora of descriptors with varying degree of computational effort for their calculation are available. Among the most commonly employed descriptors in organocatalysis are steric and electronic descriptors. Section 2.1 provides a detailed overview of examples where different kind of descriptors have been successfully applied for predictive modelling in organocatalysis. In contrast to the representations through graphs, or SMILES, which can be directly obtained from the molecular structure, the selection of appropriate descriptors is problem-specific and requires knowledge about the fundamental interactions governing the reaction outcome. Hence, making the selection of input features a key step for successful modelling [58–63].

#### 1.3 Modelling

The third important requirement for building a predictive model is the model architecture. Generally, ML algorithms can be divided into reinforced, unsupervised and supervised learning. In reinforcement learning, an agent is trained to make decisions by interacting with an environment, receiving feedback in the form of rewards or penalties, and adjusting its behaviour to maximise cumulative rewards over time [64].

While reinforcement learning has not yet found widespread application in organocatalysis, supervised and unsupervised learning are widely employed techniques. The latter uses unlabelled data (e.g., data without a label or numerical value), to identify patterns and relationships within the provided data. Popular tools are Principal Component Analysis (PCA), Uniform Manifold Approximation and Projection (UMAP) [65], or t-distributed Stochastic Neighbour Embedding (t-SNE) [66], which have found application in organocatalysis to reduce the dimension of the respective reaction space, e.g., for visualization purposes. Another widely applied unsupervised ML technique is clustering, which aims to group similar data points together and thus enables a diverse selection by uniformly sampling from the created space [67–68]. Supervised learning requires labelled data and aims at identifying correlations between the target values and the corresponding input features. In the context of addressing chemical problems, this can be used to correlate reaction specific features with the reaction outcome, such as the yield or selectivity. A plethora of different supervised learning algorithms are available and a priori knowledge which architecture works best is challenging. Some of the most widely used algorithms include multivariate linear regression (MLR) [69] in which the target is linearly modelled by multiple independent variables. Other notable architectures include decision trees [70], support vector machines [67] and deep neural networks [71–72]. While the accuracy of the model is paramount, interpretability is also highly desirable. In this regard, MLR bears the advantage that it yields a directly interpretable function which can be used for mechanistic inference. However, it is important to note that the caveat of correlation and causality must be considered. Also, for other kind of models, e.g., random forests, it is common practice to consider the importance of individual features for the model’s prediction to gain mechanistic insight. Careful attention must be paid to the collinearity of features [73], such that they are not too strongly related to each other, which complicates any quantitative interpretation of feature importance. Thus, thorough analysis and special strategies to address collinearity, such as hierarchical clustering [74] or threshold-based pre-selection [75] have to be considered to ensure reliable interpretability [69].

It is worth mentioning that all the above-mentioned techniques are not limited to applications in organocatalysis but are used for a wide variety of chemical problems.

### 2 ML for selectivity predictions

**Figure 3:**
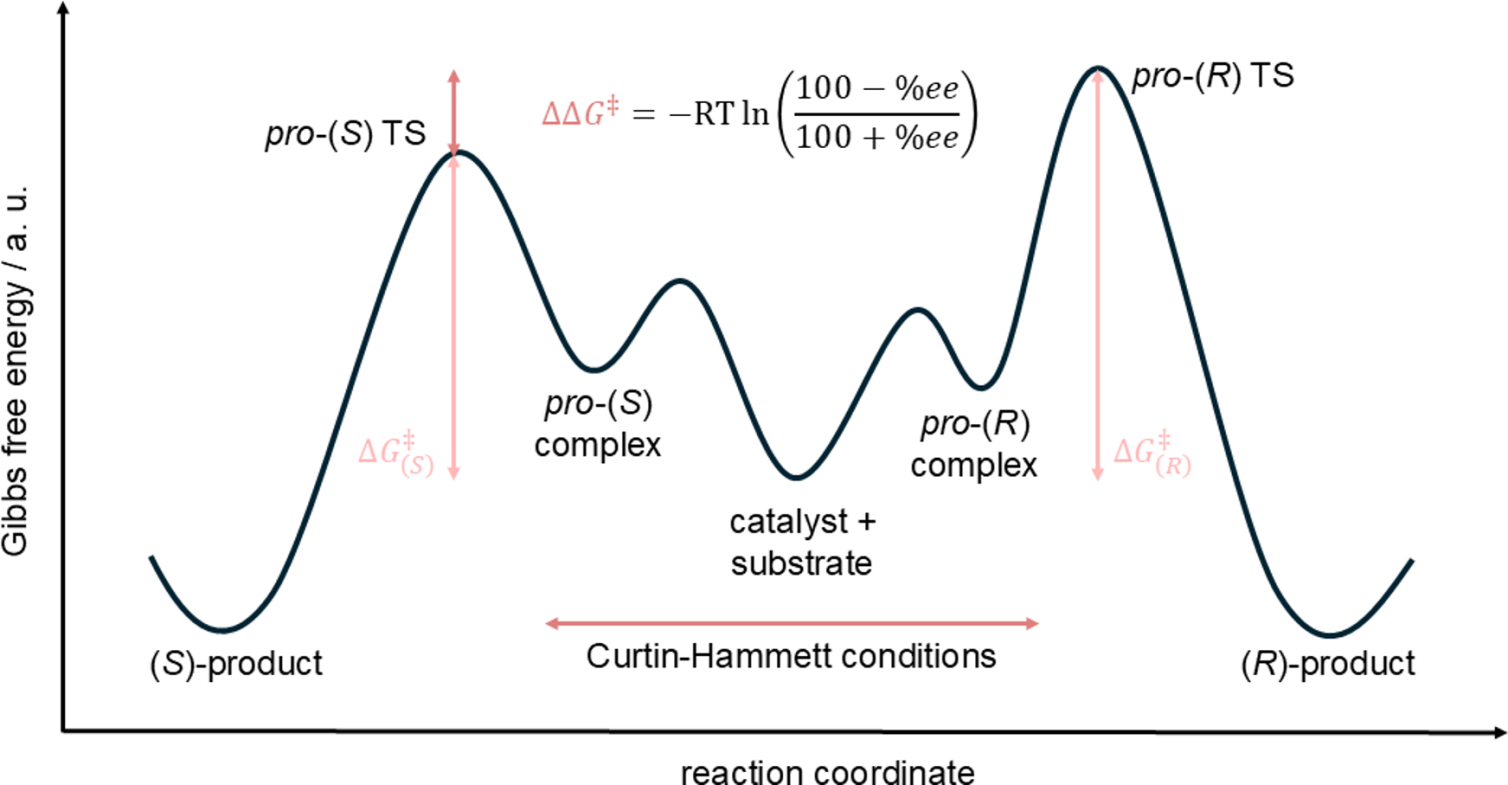
Depiction of the energy diagram of a generic enantioselective reaction. In the centre, catalyst and substrate are separated. They associate with each other to either the pro-(*R*) or pro-(*S*) complex, with all these reactions taking place in a fast equilibrium (Curtin–Hammett conditions). From these complexes, the products are formed via separate transition states. The energy difference between these two transition states is termed ∆∆*G*^‡^ and determines the selectivity.

In the context of organocatalysis, for a majority of published work, the reaction property of interest is the selectivity (either enantio- or diastereoselectivity), which is predicted as the difference in energies between the selectivity-governing transition states ∆∆*G*^‡^ (Figure 3).

**Figure 4:**
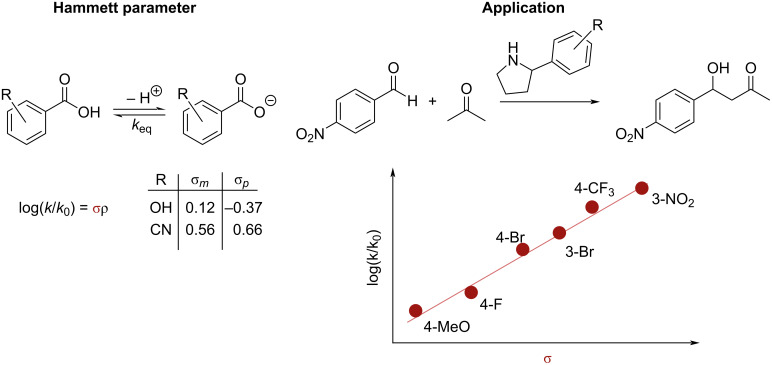
Hammett parameters are derived from the equilibrium constant of substituted benzoic acids (example from Rogers et al. [76] to correlate Hammett parameters of the arylpyrrolidine catalysts to the reaction kinetics of the aldol reaction).

Whereas the application of the above described representations and models to such problems is rather modern, the interest to describe the influence of substrate or catalyst structures on the rate or selectivity of a reaction is well-established and led among others to the introduction of Hammett parameters to relate chemical structures to both kinetic and thermodynamic reaction properties [28] (Figure 4).

As Hammett parameters account only for the electronic effect of substituents, much research has been devoted to develop physical-organic descriptors, which consider steric effects and separate the electronic effect into contributions from resonance and induction, among others [27,77–81].

In this chapter, we first discuss the evolution of physical-organic descriptors for the representation of organocatalysts [82]. Later, we examine the effects of increasing data availability towards the application of ML in this field.

#### 2.1 Evolution of physical-organic descriptors in organocatalysis

Drawing inspiration from linear free energy relationships, MLR models, pioneered by Norrby and co-workers [83] and later further developed by Sigman and co-workers [69,82], are commonly used for the prediction of enantioselectivity. In such models, the substrates, catalysts, and other relevant reaction species are encoded via a suitable representation of expert-chosen descriptors. Subsequently, the target property of interest, commonly ∆∆*G*^‡^, is fitted to the representation via a linear fit of the form *y* = *m*_1_*x*_1_ + *m*_2_*x*_2_ +…+ *m_n_x_n_* + *k*, where *y* is the target property, *m*_1_, ... , *m_n_* are the regression coefficients, *k* is the offset and *x*_1_, …, *x_n_* are the molecular descriptors. The regression coefficients are also indicative of the importance of the respective molecular parameter. Thus, MLR models provide the capability to directly interpret the prediction results and form mechanistic hypotheses based on the importance of distinct descriptors.

**Figure 5:**
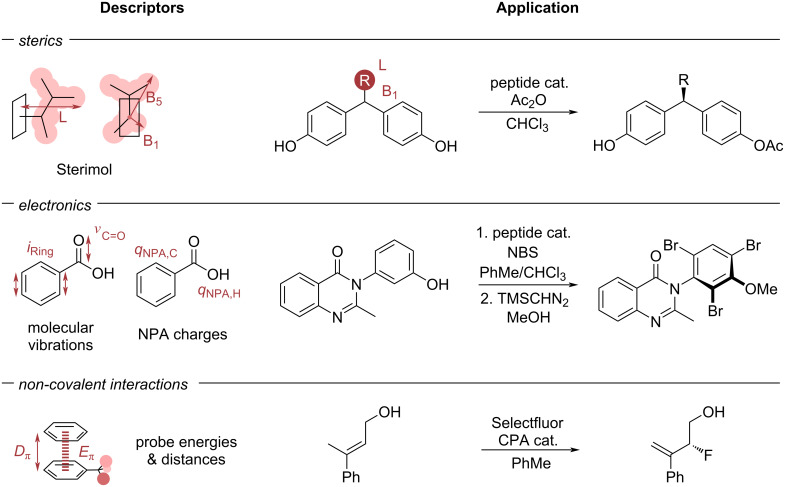
Selected examples of popular descriptors applied to model organocatalytic reactions. Descriptors encompass steric features modelled via Sterimol parameters [84] (example from Harper et al. [60] correlating the Sterimol B1 and L parameters of the bisphenols to the enantioselectivity of the peptide catalysed desymmetrisation), electronic features modelled via vibrations or NPA charges (example from Crawford et al. [86]) and NCIs, modelled via interaction distances and energies with a defined probe (example from Orlandi et al. [61]).

Given the importance of the chosen representation, the search for descriptive parameters has always been a cornerstone in this field. While Taft [77] and Charton [81] describe steric properties as singular substituent values, Harper et al. [60] showed that a singular value is insufficient to represent steric substituent properties. Instead, the authors used Sterimol parameters [84] as steric descriptors (Figure 5), showing superior correlations towards the enantioselectivity for a multitude of organocatalytic reactions.

Sterimol parameters are calculated from a given 3D structure and consist of three parameters, describing the minimum and maximum (rotational) width as well as the depth of a substituent. Nowadays, Sterimol parameters are established as standard parameters to describe steric residue properties. Since Sterimol parameters are calculated from a 3D structure, it is important to include information from relevant conformers. To avoid losing important information from discarding conformers, Paton and co-workers [85] introduced wSterimol, which takes into account structures from the entire conformer ensemble via Boltzmann-weighting. The authors used their descriptors for the prediction of the enantioselectivity for several previously reported reactions, showing improved prediction performance compared to non-Boltzmann-weighted Sterimol parameters. Apart from considering parameters of the entire conformer ensemble, it has been shown that informative models can be developed by considering active structures. This was demonstrated by Crawford et al. [86] in their investigation of a peptide-catalysed atroposelective bromination (Figure 5). The authors found that the peptidic catalysts can broadly be defined in two categories of β-turns: a type I’ pre-helical and type II’ β-hairpin. Even though the latter was consistently lower in ground state energy (up to 6 kcal/mol for some catalysts), predictive models for enantioselectivity were found for both catalyst conformers in separate MLR models. For organophosphorous ligands of transition metal complexes, the minimum buried volume in a conformer ensemble was identified to determine the ligation state towards a metal centre as either mono- or bis-ligated and thus providing a threshold for catalytically active ligands [87]. All of these examples demonstrate that not only the type of descriptor is important, but also the structure for which the descriptors are considered. This can either be ensured by expert-knowledge of preselecting relevant structures, for example based on a known mechanism, or by considering information from the entire conformer ensemble.

Parallel to the evolution in modelling steric effects, the representation of electronic effects has also been further developed. Milo et al. [58] introduced the intensity and frequency of manually selected molecular vibrations as descriptors (Figure 5). For the selection of relevant vibrations, a mechanistic proposal is required a priori, commonly based on a manual analysis of the probed substrates. The inclusion of electronic parameters led to a considerable improvement in predicting the enantioselectivity of a peptide-catalysed bisphenol desymmetrisation compared to their omission, showcasing the importance of capturing relevant molecular properties via descriptors. Apart from molecular vibrations, electronic influences are commonly modelled via global properties of a molecule (such as HOMO/LUMO energies) or local properties (such as natural population analysis (NPA) charges/NMR shifts), as shown in Figure 5 [69,72,88–89].

With respect to organocatalysis, NCIs are often a major factor in determining selectivities, which are hard to describe via standard molecular descriptors. Therefore, Orlandi et al. [61] introduced computed NCI distances and energies between benzene and a probe residue as descriptors for NCIs (Figure 5).

Notably, the NCI energies are inspired by previous work from Wheeler and Houk [90–91] and are defined as the computed energetic difference between the complex of the benzene ring and the probe residue and the separated species. Orlandi et al. used the NCI parameters in combination with other descriptors to model the enantioselectivities of a kinetic resolution of benzyl alcohols and an enantiodivergent fluorination of allylic alcohols, observing good correlations for both reactions. Since then, the proposed NCI descriptors have been successfully applied to multiple different reactions, such as an allenoate Claisen rearrangement [92] and a phase-transfer catalysed oxidative amination reaction [93]. In the latter, NCI descriptors were both used to simplify previously existing MLR models and also led to a hypothesis of key NCIs in the transition state. Whereas these descriptors require the selection of a suitable probe model, Chen and Pollice proposed P_int_ as a descriptor of the London dispersion potential that is universal and can be calculated without a probe system [94]. Although P_int_ has not been utilised for organocatalysis, the authors applied it to a Pd-metal-catalysed enantioselective 1,1-diarylation of benzyl acrylates [95] and found a similar performance compared to NCI probe descriptors.

**Figure 6:**
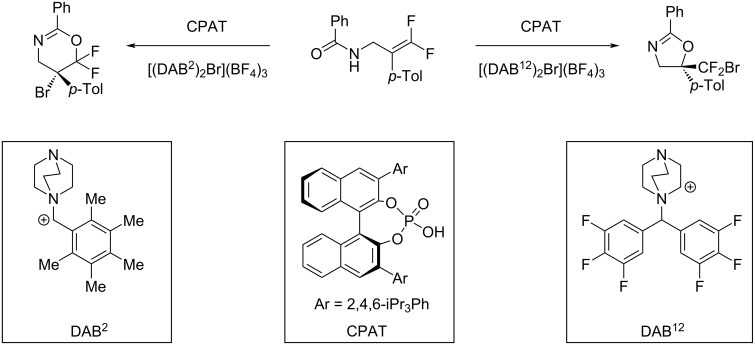
Example bromocyclization reaction from Toste and co-workers using a DABCOnium catalyst system and CPA phase transfer catalyst [96].

Despite the success of this approach, it is important to remember that descriptors do not have to be parameters of one molecule and that intermolecular terms can be used to derive mechanistic hypotheses. Toste and co-workers [96] investigated a bromocyclization catalysed by a chiral phosphoric acid (CPA) and a DABCOnium brominating reagent (Figure 6). The authors calculated transition state conformer ensembles for several flexible DABCOnium systems and performed energy decomposition analysis to separate the interactions between catalyst, substrate and the DABCOnium moiety. Subsequently, a random forest model was used to predict *exo*/*endo*- and regioselectivity of the reaction. Using random forest as an interpretable machine learning model allowed to extract the important features of the model, which indicated that the dispersion interaction between the DABCOnium system and the CPA is governing the *exo*-selectivity.

**Figure 7:**
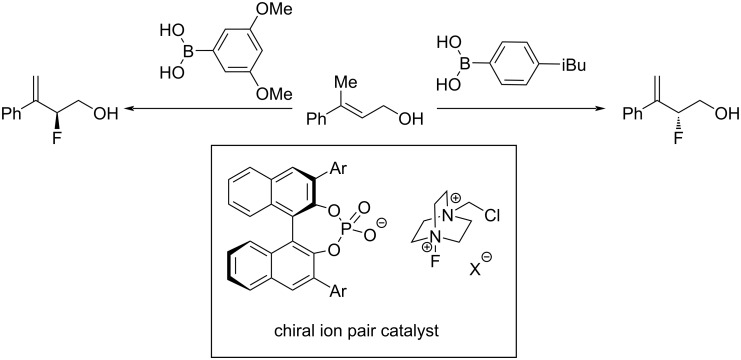
Example from Neel et al. using a chiral ion pair catalyst for the selective fluorination of allylic alcohols [97].

For the application of the ML techniques discussed above, it is assumed that all studied reactions follow the same mechanism. If that is not the case, models cannot be reliably fit to the data points, similar to mechanistic breaks in Hammett plots. However, deliberate data set design to systematically cover the relevant chemical space can aid in detecting outliers and aid in creating more relevant models, as demonstrated by Neel et al. for an enantiodivergent fluorination of allylic alcohols, catalysed by a CPA as phase transfer catalyst and an arylboronic acid [97] (Figure 7).

After a systematic data set design involving eight phosphoric acids and eight boronic acids, the authors observed breaks in linearity of the model of enantioinduction for some catalyst combinations. Further experiments, such as non-linear effect studies and isotopic substitution experiments revealed multiple different mechanisms of enantioinduction for the respective combinations. To rationalise relevant interactions, MLR models were trained on subsets of the data set. For each different mechanism of enantioinduction previously elucidated, the authors developed a separate model to gain a sufficiently interpretable model, finding that some parameters remain important throughout the different subsets. This example demonstrates both the strength of careful data analysis and the intricacies of dealing with chemical reactivity data.

The above outlined examples demonstrate the relevance of efficient representations, to which the development of advanced descriptors contributed. However, the usage of descriptors also restricts the generalizability of models, as they have to be expert derived. Interestingly, descriptor-based MLR models have also been used to predict the Mayr–Patz nucleophilicity parameter N, which estimates the nucleophilicity of a nucleophile based on experimentally measured kinetic data. The MLR models are used to predict N for more than 1200 nucleophiles, enabling the prediction of N for further nucleophiles [98–101]. While this complicates the usage of descriptors for a multitude of different reactions, it also enables an efficient representation by representing chemical hypotheses. Even though descriptors have been proposed for a number of different interactions, others are not easily represented via descriptors but remain highly important towards enantioselectivity, e.g., solvent-solute interactions.

When interpreting the importance of descriptors, effects such as overfitting and collinearity of features must be accounted for. Particularly in the low-data regime, the importance of selected features can vary based on the reactions that are contained in the training and test set. While descriptors can help in gaining mechanistic insight, it is important to not overinterpret the significance of single features to form a mechanistic hypothesis.

Ideally, to overcome issues such as a high dataset dependence, larger reaction datasets are available. In terms of data set sizes, the presented studies all worked in the low to medium data set size, with up to few hundred experiments [102–103], where careful considerations must be paid towards the applicability domain, overfitting and interpretability. With HTE platforms established and due to their importance to ML campaigns, the past few years have seen a trend in creating larger experimental chemical reactivity datasets, in particular for transition metal catalysis [47–48].

#### 2.2 Increasing data availability in ML for organocatalysis

**Figure 8:**
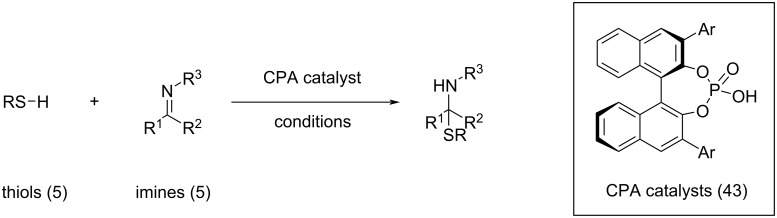
Data set created by Denmark and co-workers for the CPA-catalysed thiol addition to *N*-acylimines [67]. The combinatorial data set encompasses the enantioselectivities from 5 thiols and 5 imines in combination with 43 CPA catalysts for a total of 1,075 data points.

While, to the best of the authors’ knowledge, no HTE dataset has found widespread application in ML for organocatalysis, Denmark and co-workers published a data set comprising more than 1,000 organocatalytic transformations [67]. In their work, the authors demonstrated a data-driven workflow to study the enantioselective formation of N,S-acetals catalysed by CPAs. To represent the catalysts, the authors developed the average steric occupancy (ASO) descriptors, a representation inspired by CoMFA [104–106], which recently also was applied in the selectivity prediction of aldehydes to nitroalkenes [68]. In ASO, all catalysts are aligned on a 3D-grid and the descriptor is calculated as the average occupancy of voxels on the 3D grid, where a voxel is occupied if it is within the van der Waals radius of an atom. The steric descriptors were combined with electronic descriptors called Average Electronic Indicator Field (AEIF), which are calculated for each CPA substituent (R) by observing the electrostatic potential of a quarternary ammonium ion with the substituent of interest (NMe_3_R^+^). The authors performed unsupervised clustering on an in silico library to select a ‘Universal Training Set’ (UTS) consisting of 24 catalysts, aiming to effectively represent the chemical space of CPAs. This UTS was selected by first reducing the dimension of the combined descriptor space using PCA and subsequent uniform sampling of the catalysts using a clustering algorithm (see Section 1.3), which ensures a broad coverage of CPA chemical space. Notably, this data-driven technique is not restricted to the reaction chosen by the authors. The UTS, combined with 19 ‘test set’ catalysts, 5 nucleophiles and 5 electrophiles, constitutes a dataset of 1,075 reactions with associated enantioselectivity values (Figure 8).

The size of the data set allowed the authors to perform various ML experiments: a random (600:475) split on the data set, a substrate test set where ∆∆*G*^‡^ of known catalysts with new substrate combinations were predicted, a catalyst test set where the substrates were known but the catalysts not and a test set were both components were not known beforehand. Even in the most challenging case, predictions were highly accurate with a mean absolute deviation of 0.24 kcal/mol. Further, the authors performed a split where the models were only trained on reactions with an ee < 80% (718:357 split), still showing good extrapolation performance with an error of only 0.33 kcal/mol on the test set with higher enantioselectivity.

**Figure 9:**
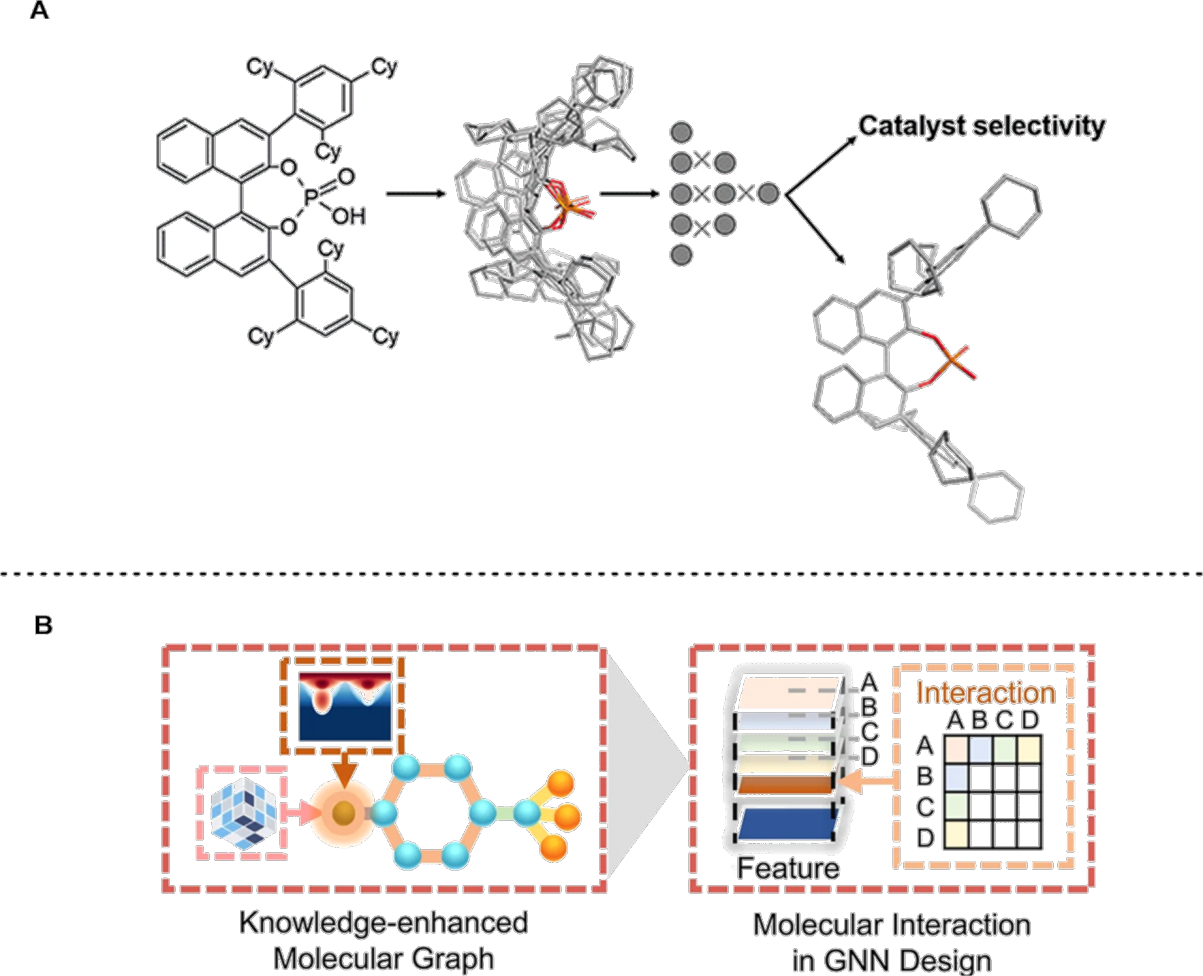
Selected examples of ML developments that used the dataset from Denmark and co-workers [67]. (A) Varnek and co-workers used ML models designed to deal with multiple catalyst conformers for the prediction of catalyst selectivity. Reproduced with permission from reference [109], © 2021 Georg Thieme Verlag KG. This content is not subject to CC BY 4.0. (B): Hong and co-workers utilised a molecular graph based on knowledge about the local steric and electronic information, coupled with a graph neural network equipped with a module designed to capture molecular interactions. Figure adapted from reference [113] (© 2023 S.-W. Li et al., published by Springer Nature, distributed under the terms of the Creative Commons Attribution 4.0 International License, https://creativecommons.org/licenses/by/4.0).

The open availability of larger, high-quality datasets also inspires other researchers to develop and apply ML algorithms and molecular representations. The previously described dataset from Denmark and co-workers has been adopted by other groups to develop and/or benchmark descriptors [107–108], models that use architectures designed to deal with multiple conformers [109–111] (see Figure 9A and also Section 2.1) or models that are based on multiple fingerprints [112].

In addition, such larger data sets also lead to an increased interest in the application of deep learning tools, such as graph-based neural networks, to organocatalysis. One particular example was published by Hong and co-workers [113], who developed a chemistry-informed graph model for the prediction of enantioselectivities (Figure 9B). In their model, molecules were represented as graphs, where local steric and electronic information was added to each node (atom). Additionally, the used graph neural network contains a molecular interaction module that allows the model to learn synergistic effects between molecules, crucial for reactivity prediction tasks. While reaching state-of-the art performance in predicting ∆∆*G*^‡^ on the data set from Denmark and co-workers, the designed neural network also enables to interpret the effects leading to the observed enantioselectivity by eliminating the atom features and observing the change in predictive performance. Using this method, the authors observed that the main contribution towards enantioinduction by CPAs is through steric effects, in line with previous literature.

Besides the establishment of experimental data sets, the number of ML data sets based on quantum mechanical calculations is also increasing, such as a data set that considers propargylation reactions catalysed by bipyridine *N*,*N*’-dioxide-derived scaffolds, created by Wheeler and co-workers using their AARON toolkit [21,114–116]. Similar to experimental data, computational data sets also lead to the development of ML innovation [117–118]. One example is the development of a new reaction representation based on the geometry of reactants and products [89]. Unlike expert-chosen descriptors, this representation is generalisable to other systems. Although not concerned with selectivity, Corminboeuf and co-workers reported OSCAR, a computational repository of 4,000 organocatalyst structures mined from the literature and Cambridge Structural Database (CSD) [31].

In addition, the authors utilised the combinatorial nature of organocatalysts to create data bases comprising more than 8,000 NHC-type catalysts and more than one million double hydrogen bond donor catalysts. While this repository does not provide any reactivity data, it still comprises a valuable map of organocatalyst chemical space to aid in catalyst design.

The creation of these larger datasets, both experimental and in silico, has enabled the interest of the ML in chemistry community towards enantioselective organocatalysis. With these datasets, it is now possible to test different algorithms and benchmark varying chemical representations. Despite these advances, the existence of few large datasets in enantioselective organocatalysis might lead to a bias in developed algorithms and representations. Since few datasets are available, advances are benchmarked on these datasets and commonly only published if they provide state-of-the-art performance. Thus, a bias towards representations and algorithms that capture relevant effects of the existing datasets are conceivable, while other important effects that govern selectivities remain underexplored by the community. Therefore, it is highly relevant to extend the available chemical space to underexplored regions and to acquire large datasets for such cases to allow for more holistic investigations of algorithms and chemical representations.

To summarise, the last decade has seen a steady refinement in the representation of chemical species, considering sterics, electronic properties and non-covalent interactions. Since these interactions are governing any reactivity, accurate description is relevant for a successful ML campaign. Most of the work in organocatalysis using expert-derived descriptors has been conducted in the low to middle data-regime. Only recently, the focus has shifted towards bigger data sets of more than 1,000 reactions, the first one of which has already inspired a manifold of other groups to develop new ML techniques, including graph neural networks. With the continued rise of high-throughput experimentation in organocatalysis [40], we expect ML to be applied to more data sets in this domain to aid in answering a wider variety of research questions. For the prediction of selectivities, we expect more advanced techniques to be adopted, establishing ML as a powerful tool for the evaluation of organocatalysts.

### 3 ML for the design of privileged organocatalysts

Throughout the development of organocatalysis, privileged catalysts, i.e., catalysts which catalyse a wide variety of different reactions through the same mechanism of enantioinduction, have emerged in multiple organocatalytic transformations [119]. The examples discussed in Section 2 all have seen the application of ML techniques to predict the selectivity of a reaction of interest. However, since the mechanism of enantioinduction is similar for multiple reactions catalysed by a privileged catalyst class, these ’related’ reactions can in principle be modelled together. The reactions are assumed to be mechanistically transferable.

The similarity of multiple reactions led to two different applications of ML to organocatalysis: (**1**) prediction of reaction properties (e.g., selectivity) for multiple mechanistically transferable reactions, and (**2**) employing ML in the search to predict the generality of a catalyst. This chapter will discuss prominent examples in both applications.

#### 3.1 ML for transferable reactions

The key to modelling transferable reactions together is to find a representation that can describe all relevant reacting species. While such representations commonly exist in chemistry, e.g., SMILES and graphs, the most common representation for transferable reactions is via expert-chosen descriptors. As such, the space of relevant reactions has to be carefully studied, e.g., with respect to the different reactant or catalyst classes. Once this space is defined, the descriptors have to be chosen such that they are specific enough to provide information to the ML model while also general enough to cover the space of interest.

One pioneering study in the field of mechanistic transferability for enantioselectivity prediction was published by Reid and Sigman [120] in 2019. The authors manually combined 367 different published reactions of BINOL-phosphoric acid catalysed nucleophilic additions to imines, comprising alcohols, thiols, phosphonates, diazoacetamides, peroxides, benzothiazolines and more as nucleophiles. Apart from reactant classes, the reactions also vary in additives, and solvent among others. Since these reactions all adhere to the same mechanism of enantioinduction, the authors chose to consider them in the same ML campaign, even though the nucleophiles vary significantly. As descriptors, the authors used the overlapping features of nucleophiles, imines and catalysts to derive steric and electronic parameters as well as topological descriptors for solvents, where less structural overlap is present [121].

**Figure 10:**
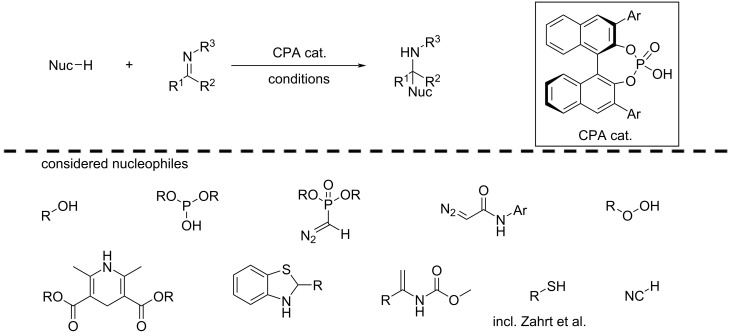
Study from Reid and Sigman developing statistical models for CPA-catalysed nucleophilic addition reactions to imines for different classes of nucleophiles [120].

For every reaction, the imine is categorised as either an *E*- or *Z*-imine, based on the sign of the recorded enantiomeric excess. Further, molecular descriptors, either physicochemical properties or topological, are calculated for all reaction partners. This data is used to develop a comprehensive model, finding that imine parameters govern the defining transition state and hence the preferred enantiomer. In a focused modelling, two separate models are constructed, one for all *E*- and *Z*-imines, respectively, finding substrate–catalyst matching is important for *E*- and *Z*-imines. The focused correlations enabled the authors to identify subtle mechanistic differences between reactions of *E*- and *Z*-imines, such as the role of steric and electronic properties of the imine for *E*- and *Z*-imines, respectively. The two-stage workflow, using the comprehensive model to distinguish the imine-type and subsequently using the focused model for detailed predictions, proved successful for out-of-sample reaction predictions with new nucleophiles, such as enecarbamates. Further, the authors also tested their models on the dataset published by Denmark and co-workers [67] (see Figure 10), showcasing the importance of high-quality datasets for ML applications.

**Figure 11:**
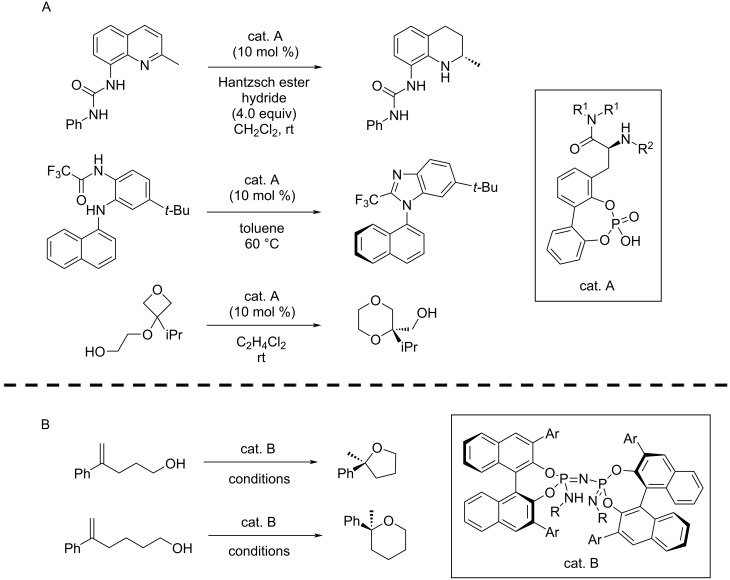
Selected examples of studies where mechanistic transferability was exploited to model multiple reactions together. (A): Liles et al. used univariate classification and MLR to develop a new CPA catalyst achieving high enantioselectivity for transfer hydrogenations. A comprehensive model for multiple reactions was developed under the assumption of mechanistic transferability [124]. (B): List and co-workers employed Support Vector Machines trained on data of different cyclisations to find an optimal catalyst for tetrahydropyran synthesis [107]. R = general residue, Ar = aromatic residue.

Due to their prominence in organocatalysis, CPAs have been a common catalyst class when considering mechanistically transferable reactions for modelling. Further work on CPA catalysed reactions was performed by Shoja et al. [122], considering a multitude of different reaction types, ranging from hydrogenations to epoxidations and dearomatization reactions. In a further study, the generalisation of the obtained model to reactions involving more complex substrates was demonstrated [123]. For the comparison of different reaction descriptors, Asahara and Miyao [108] considered different CPA-catalysed nucleophilic additions to imines, comprising aza-Mannich reactions and Friedel–Crafts reactions among others. Different reactions were also combined by Liles et al. [124]. For a transfer hydrogenation reaction, the authors used a workflow consisting of training set design, classification, MLR and extrapolation to predict a new class of CPA catalysts with enhanced enantioselectivity. Subsequently, the new catalyst class was tested for cyclodehydration and oxetane desymmetrisation reactions, where a comprehensive model was developed for the three different reactions (Figure 11A).

Mechanistic model transferability for CPA-catalysed Minisci reactions [125] was utilised for the derivatization of quinolines and pyridines. Models trained on these compound classes show good generalisation towards other nitrogen-containing heteroaromatics including pyrimidines and pyrazines.

The importance of mechanistic understanding for model building was underlined by Kuang et al. [126], where the authors considered multi-catalyst enantioselective reactions, where one catalyst was an organocatalyst, either CPA or an amine. The co-catalyst was included in the ML model by being considered as a nucleophile or electrophile, depending on the reaction mechanism. Descriptors allowed for the inclusion of a variety of co-catalysts, ranging from Fe-piano stool complexes to copper complexes. The consideration of co-catalysis into model development further expands the considerable reaction space in organocatalysis.

The discussed principle of mechanistic transferability has also been employed outside of CPA catalysis, with a focus on amine-based hydrogen-bond donors, for example imidodiphosphorimidate-type catalysts for the construction of THF and THP rings [107] (Figure 11B). Werth and Sigman [127] investigated multiple nucleophilic additions to nitroalkenes, catalysed by bifunctional hydrogen bond donors, observing good correlations to new bi-functional donors, new nucleophiles, new electrophiles and even similar cascade-type reactions.

In the authors’ perspective, the exploitation of the concept of mechanistic transferability is a promising avenue for the application of ML in enantioselective organocatalysis, as combining data from multiple reactions enlarges datasets. As such, it is an important stepping stone towards the development of more generally applicable models. However, when applying these models, potential mechanistic breaks as well as utility of the chosen representations (descriptors) across the entire dataset have to be considered. Currently, the work mainly focuses on CPAs for which a vast number of reactions are reported. While this underlines the importance of CPAs as enantioselective organocatalysts, work exploring the mechanistic transferability of other catalyst classes should not be neglected in order to fulfill the potential that the application of ML in organocatalysis holds.

#### 3.2 ML for general organocatalysts

While it is important to consider catalysts achieving high enantiomeric excess (ee) on relevant reactions, the deployment of general catalysts that provide a reasonable ee for a variety of reactions has gained more attention over the last years [128–130]. Catalysts that fulfil such demands are coined ‘general catalysts’.

While the concept of generality was recently explored in a closed-loop fashion for Suzuki–Miyaura cross couplings to find the most general catalyst and reaction conditions [131], the application of this concept in the context of ML has found comparatively less attention in organocatalysis, despite the prominence of privileged catalysts.

Despite the intuitive explanation of generality to chemists, a clear mathematical definition of chemical generality remains elusive, exacerbating the integration of the generality concept towards machine learning algorithms. As such, different implementations were chosen to tackle this problem.

**Figure 12:**
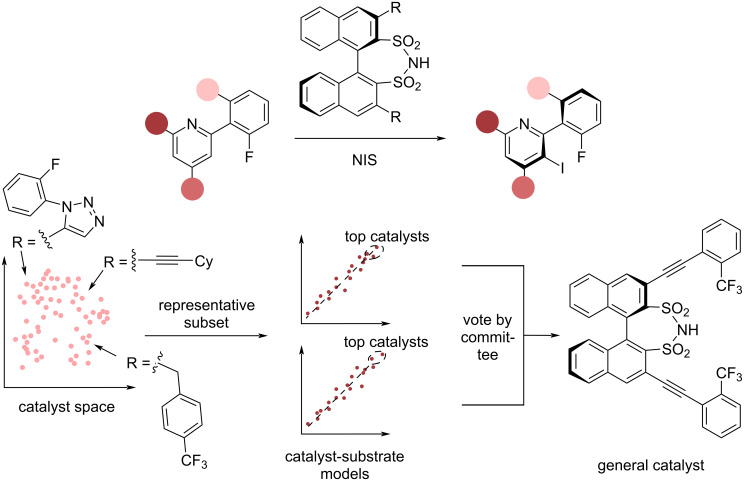
Generality approach by Denmark and co-workers [132] for the iodination of arylpyridines. From the relevant chemical space, a representative subset of 18 catalysts is selected. For each of the 13 model substrates, a catalyst-substrate model is trained. Catalysts that are top performers for multiple substrates are considered general catalysts.

In 2022, Denmark and co-workers [132] (Figure 12) investigated a disulfonimide-catalysed atroposelective iodination with the intention of finding a general reaction procedure. After constructing an in silico library consisting of 1,478 catalysts, a universal training set was constructed consisting of 18 catalysts. Subsequently, the enantioselectivity of each catalyst with 13 model substrates was experimentally evaluated. 13 different models, one for each substrate, were developed. To find a general catalyst, a technique termed ’catalyst selection by committee’ (CSC) was employed: for each substrate, all in silico catalysts were evaluated and catalysts in the most enantioselective 1% of catalysts considered received one ’vote’. After this process was performed for each of the 13 model substrates, catalysts with more votes were termed as being more general, balancing high enantioselectivity with a broader substrate scope. CSC enabled the identification of two well-performing, general catalysts.

**Figure 13:**
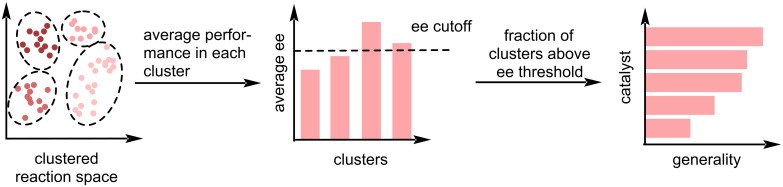
Betinol et al. [133] clustered the relevant chemical space and then evaluated the average ee for every cluster. Catalysts that perform above a user-defined ee cutoff for many clusters are considered general catalysts.

A different generality metric was proposed by Betinol et al. [133] (Figure 13). The authors performed clustering on the reaction space of interest representing the molecule either by topological or quantum mechanical descriptors. The generality of a catalyst was then assigned by considering the fraction of clusters for which the average cluster enantioselectivity of a catalyst exceeds a user-defined threshold. This threshold can be used to balance the need for a wide substrate scope and enantioselectivity requirements, while accounting for the specifics of a reaction and the requirements of the user. The authors applied their method on 3,003 literature-mined Mannich reactions from 106 publications to find that urea-based catalysts are the most general organocatalysts for this reaction class (ee threshold 80%), even though amine-based catalysts demonstrate a higher average ee. Notably, this strategy is not restricted to literature-extracted examples and can also be applied to enantioselectivities calculated via quantum chemical calculations or predictions from an ML model. The latter was used by the authors as an augmentation technique towards an imbalanced dataset for CPA-catalysed nucleophilic additions. Further, the authors also propose an order of generality for CPAs catalysing nucleophilic additions to imines, with TRIP being the highest ranked (ee threshold 60%). Thus, the authors recommend that for developing a new reaction, their metric can be used to decide which catalyst should be tested first based on the expected success. This generality-based guiding principle of experimental design showcases a further possibility for data-driven methods to complement and augment experimental chemistry.

**Figure 14:**
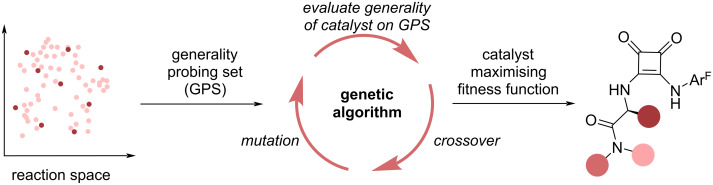
Corminboeuf and co-workers [134] chose a representative subset of the reaction space (indicated by dark red points) and used it to evaluate the generality of catalyst structures optimised through their genetic algorithm.

In addition to these methods, Corminboeuf and co-workers [134] proposed a genetic algorithm for the de novo design of general catalysts (Figure 14). Considering the Pictet–Spengler cyclization of tryptamine derivatives catalysed by hydrogen-bond donors, the authors considered a general catalyst to display both high enantioselectivity and turn-over frequency for a broad substrate scope. The substrate scope, termed generality probing set (GPS), was selected based on farthest point sampling of a literature mined reaction space to cover a wide chemical space. To assess the enantioselectivity and turn-over frequency for reactions with a new catalyst, which is required for de novo design, the authors used different strategies. To predict enantioselectivity of a previously unseen reaction, the authors used the reported enantioselectivities in their initial literature-mined reaction database to train an XGBoost model. The turn-over frequency of a reaction was determined using a volcano plot based on reaction energies [135–137], where the latter were again predicted using an XGBoost model based on the literature-mined dataset. Using fragments derived from their OSCAR [31] database, the authors used the NaviCatGA genetic algorithm [118] to find the most general catalysts. The fitness function comprised multiple objectives, including the median of the enantioselectivity and activity across the generality probing set. The usage of a multi-objective optimization algorithm allowed them to discover multiple trade-off optima, enabling a scientist to select the ideal catalyst based on the specific requirements of catalytic activity and selectivity, while still accounting for catalyst generality through design of the objectives. Noticeably, data analysis allowed to identify regions in the chemical space where highly ranked catalysts underperform as well as less sensitive areas in chemical space, further providing mechanistic insight into the mechanism of stereoinduction and activity trends.

With the concept of privileged catalysts deeply rooted in organocatalysis, we expect a steady increase in studies aiming to bridge the gaps between different reactions that are mechanistically transferable via ML. Using this strategy, it is possible to both increase the available data (since more reactions are considered), as well as investigate more general mechanisms. However, careful consideration has to be paid towards combining different reactions, as mechanistic transferability has to be ensured. Furthermore, the usage of ML to identify general catalysts demonstrate that the application of modern ML tools is not limited to predicting selective catalysts.

### 4 ML for catalyst and reaction design

The design of chemical reactions encompasses various aspects, from the choice of the employed catalyst to the selection of ideal reaction conditions. While traditionally, all of this has been performed by chemical knowledge, intuition and rational design, the last years have witnessed a surge in data-driven approaches to improve the design of reactions, e.g., by inferring mechanistic features through statistical modelling, the generation of catalyst structures with increased catalytic activity, or optimising the reaction conditions to maximise the yield or selectivity. In contrast to the direct approach as seen in many examples discussed so far, where starting from a molecular structure and a set of conditions, the reaction outcome is predicted, optimising the design of a reaction can be framed as an inverse design approach [138]. Given a target, e.g., fast conversion or high selectivity, the task is to find a catalyst structure or a set of conditions to satisfy the requirement. The following chapter will give an overview of recent advances in the design of organocatalytic reactions.

#### 4.1 Mechanistic understanding

**Figure 15:**
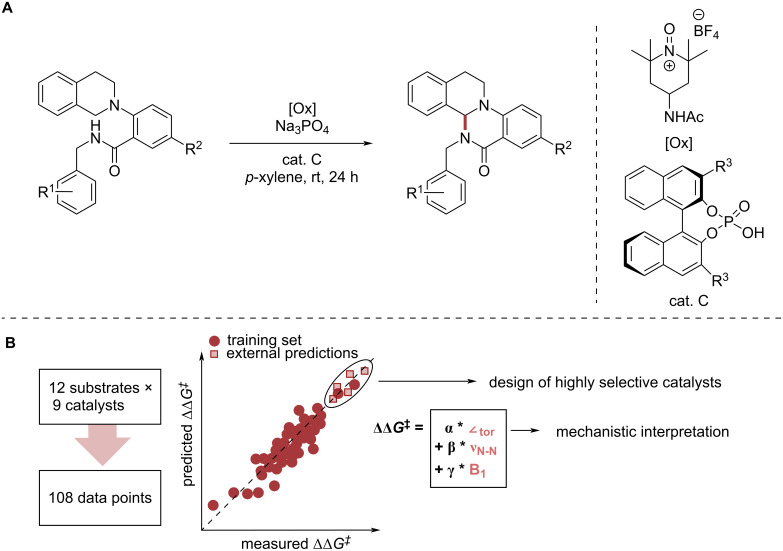
Example for data-driven modelling to improve substrate and catalyst design. (A) C–N coupling catalysed by CPA derivatives studied by Sigman and co-workers [143]. (B) The library for the study consisted of 12 substrates and 9 triazolyl catalysts. This data was used to train an MLR model and infer key mechanistic features as well as the design of highly selective catalysts which were experimentally verified after prediction.

The design of a catalyst requires detailed understanding of the key catalytic steps [23,139–142] and commonly uses calculated or measured physical parameters of reaction components to make decisions in a design effort. In line with the early developments of statistical modelling through Hammett parameters to correlate substrate properties to kinetic properties of the reaction (Section 2), advanced ML tools can help to unravel key mechanistic features in higher dimensions and with stronger interactions, which can be used to tailor a reaction to match desired properties. Sigman and co-workers demonstrated this by complementing knowledge from physical organic chemistry with data-driven analysis techniques, in particular MLR, to gain a greater understanding of the enantioselectivity-determining steps for a C–N coupling catalysed by CPA derivatives (Figure 15A) [143]. Based on their findings that π–π interactions between the catalyst’s triazole substituent and the substrate is key for stereoinduction, they designed new catalyst structures maximising the predicted selectivity. The predictions were experimentally validated confirming that their model can be used to guide the design of highly selective catalysts (Figure 15B).

#### 4.2 High-throughput virtual screening

Although such approaches showcase the ability of ML models to unravel structure–activity relationships and thereby guide the development of catalysts, the design of new structures remains influenced by the prevailing design principles of chemists. In this regard, approaches to explore uncharted regions of chemical space in a more unbiased way can help to identify previously unknown structures that exhibit desired properties. The advent of statistical models that can predict key catalytic properties has enabled pipelines to assess a great number of candidates in high-throughput virtual screening approaches [107,144–147]. Thereby, experimental efforts can be focused on the most promising candidates predicted by the model. Denmark and co-workers utilised such an approach to design highly selective catalysts for a peptide-catalysed annulation reaction [68]. Using conformer-dependent steric and electronic descriptors, they built a universal training set (UTS) consisting of 161 tripeptide catalysts. Based on models trained on the UTS they were able to identify highly selective tripeptide catalysts from a virtual library containing more than 30,000 structures. Remarkably, the predicted peptide catalysts did not follow the prevailing design principles of experimentally optimised peptide catalysts, demonstrating how ML can help to explore novel classes of catalysts. While high-throughput screening campaigns can be powerful tools for the discovery of novel structures with desired properties, their scope can be limited due to the effort associated with computing the descriptors for each individual molecule. Corminboeuf and co-workers utilised a fragment-based approach exploiting the modularity of commonly used organocatalysts. By considering individual contributions of catalyst fragments, they were able to build a combinatorial library of catalysts and predicted novel catalysts with increased reactivity for an organocatalysed Diels–Alder reaction [148].

#### 4.3 Genetic algorithms

**Figure 16:**
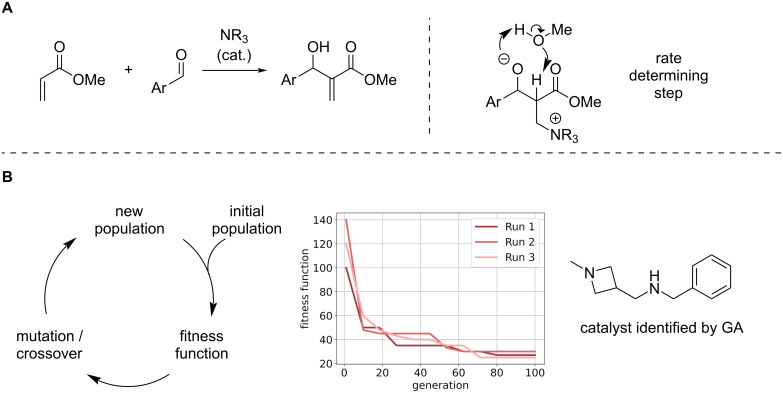
Example for utilising a genetic algorithm for catalyst design. (A) Morita–Baylis–Hillman reaction studied by Jensen and co-workers [150] (B) Left: A genetic algorithm performs mutation and crossover operations on a set of catalysts with the goal to optimise the fitness function. Middle: Schematic depiction how the fitness is iteratively optimised across multiple runs ("generations") of the genetic cycle. Right: Identified catalyst structure with increased catalytic activity.

An alternative approach for chemical space exploration is the use of genetic algorithms (GAs) [149]. Inspired by biological evolution, they aim to maximise a fitness function using biology-inspired operations such as mutation and crossovers. Jensen and co-workers demonstrated the utility of GAs by optimising the structure of a tertiary amine catalyst for the Morita–Baylis–Hillman reaction [150] (Figure 16).

First, the rate determining step was identified (within the proposed reaction mechanism). Then, the organocatalyst's structure was optimised to decrease the barrier of this step. After identification of the most potent structures by the GA, they verified experimentally that the identified structure increases the reaction rate by a factor of 7.8 compared to the commonly used DABCO catalyst. While this clearly demonstrates the capabilities of the GA to accelerate the discovery of organocatalysts, the authors note that the success of their approach is dependent on the detailed knowledge of the underlying mechanism. Therefore, the discovery of catalysts for novel reaction mechanism is still an ongoing challenge [151–153]. In order to make GAs for catalyst discovery more generally available, the Corminboeuf group developed the software suit 'NaviCatGA' [118] which is designed for the optimisation of catalysts with desired catalytic properties. The tool provides the user with considerable flexibility, e.g., the definition of the employed fitness function or the genetic operations to be applied. Further, it supports the multi-objective optimisation based on multiple target properties, which is of particular importance as an ideal catalyst combines a number of properties that need to be taken into account, e.g., solubility, stability and synthesisability. The authors exemplify this by optimising simultaneously for catalytic activity and selectivity using two individual MLR expressions in their fitness function. Doing so, their algorithm is able to tailor the structure of the employed base for a Lewis-base catalysed enantioselective propargylation of benzaldehyde in this multi-objective optimisation task [118].

Importantly, molecules designed by generative models need to be tested experimentally. This allows one to verify the assumptions made during modelling and validate the model’s ability to propose molecules tailored to a given application. In this regard, the synthesisability of the generated molecules plays a decisive role and remains a major bottleneck which currently restricts the effective use of generative models [154].

#### 4.4 ML-driven experimental design

**Figure 17:**
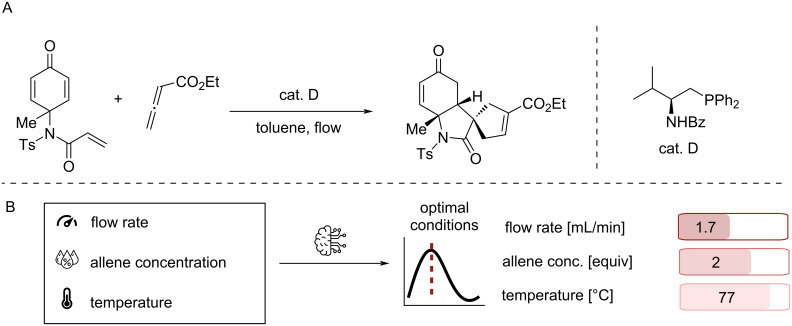
Organocatalysed synthesis of spirooxindole analogues by Kondo et al. [171] (A) Reaction scheme of dienones with allenoates to form chiral spirooxindole analogues using a chiral amine organocatalyst. (B) Schematic depiction of the employed optimisation to identify optimal conditions for flow reaction using Gaussian Process Regression. Icon ‘Flow rate’ made by Gregor Cresnar from flaticon.com. This content is not subject to CC BY 4.0. Icon ‘Allene concentration’ made by Nadiinko from flaticon.com. This content is not subject to CC BY 4.0. Icon ‘Temperature’ made by hirschwolf from flaticon.com. This content is not subject to CC BY 4.0. Icon ‘ML model’ made by VectorPortal from flaticon.com. This content is not subject to CC BY 4.0.

Besides the design of employed catalysts, reaction design involves the identification of optimal reaction conditions, which poses a formidable challenge due to the high dimensionality of the reaction space. In the simplest approach, ideal reaction conditions are identified by changing one parameter at a time based on the chemist’s intuition. While this shows the influence of the varied parameter on the observable, interaction effects between the parameters are significantly harder to capture with this approach. Design of experiments (DoE) is a more systematic approach where parameters are varied simultaneously to unravel their effect on the outcome [155–156]. Although multiple variants of DoE are available, the number of required experiments can quickly exceed what is feasible for most applications. Driven by optimisation problems in other fields, like ML model parameters, more efficient optimisation strategies have therefore been explored recently. Particularly Bayesian optimisation is widely used for optimisation problems where the quantity of interest is expensive to obtain, such as quantifying the yield of a reaction. Therefore, it has found application for the optimisation of chemical problems [157–167] and demonstrated its effectiveness by outperforming human optimisation strategies [168]. However, even with efficient optimisation algorithms, conducting experiments and analysing the reaction outcome remains a major bottleneck. Performing chemistry in flow provides several advantages in this regard, as reaction parameters can be varied on-the-fly [169]. In combination with ML optimisation strategies, this can lead to efficient optimisation of reaction conditions as demonstrated by Kondo et al. where they utilised Gaussian Process Regression (GPR) [170] to optimise the flow rate, the temperature as well as the stoichiometry of the reactant for the organocatalysed synthesis of spirooxindole analogues [171] (Figure 17).

In a later study the same group expanded the search space for a Brønsted acid-catalysed cross-coupling for the synthesis of biaryl compounds [172]. They utilised Bayesian optimisation to explore a total of six numerical and categorical parameters. With as little as 15 data points they were able to find optimal conditions which yielded the desired product in 96% yield. This showcases the application of ML-driven optimisation strategies for efficient multi-parameter screening problems, however, manual action is still required for experimental setup and analysis. Automating these operations would significantly increase productivity and reproducibility and is a research area of high interest termed self-driving laboratories [173–174]. Cooper and co-workers exemplified the opportunities of a self-driving laboratory by utilising a free-roaming robot that autonomously conducted and analysed 688 experiments selected by a Bayesian optimisation algorithm [175]. Within eight days it discovered a set of parameters that yielded a six-fold increase of activity for the photocatalytic hydrogen evolution from water compared to the baseline formulation. These examples show the possibilities that ML offers for optimising experimental design in organocatalysis. However, the use of data-driven methods to optimise reactions is still far from routine. It is expected that the recent surge of Large Language Models (LLMs) will support this development and further improve accessibility and the interaction between humans and ML-based models [176–178]. While the works presented give a glimpse of what is possible with automated experimentation pipelines in combination with ML, the wide adoption of such methods is limited by the high acquisition costs of the setup, the expertise and time required to implement and maintain the hardware in the research environment and the limited versatility of the methods to a broad range of problems [179].

## Conclusion

The tremendous potential of utilising ML tools to support organocatalysis is clearly demonstrated in the above presented works. Nevertheless, it remains to be seen whether these examples provide general solutions and are applicable to a wide range of problems. In this regard, the domain of applicability needs to be carefully analysed in order to obtain reliable and robust predictions [180–181]. While some works exemplified the ability of data-driven models to provide interpretable results, their validity is far from being universally applicable. It should be remembered that correlations in statistical models don’t equal causation, and that hypotheses made from feature importances need to be followed up by mechanistic studies to avoid potentially misleading conclusions.

**Figure 18:**
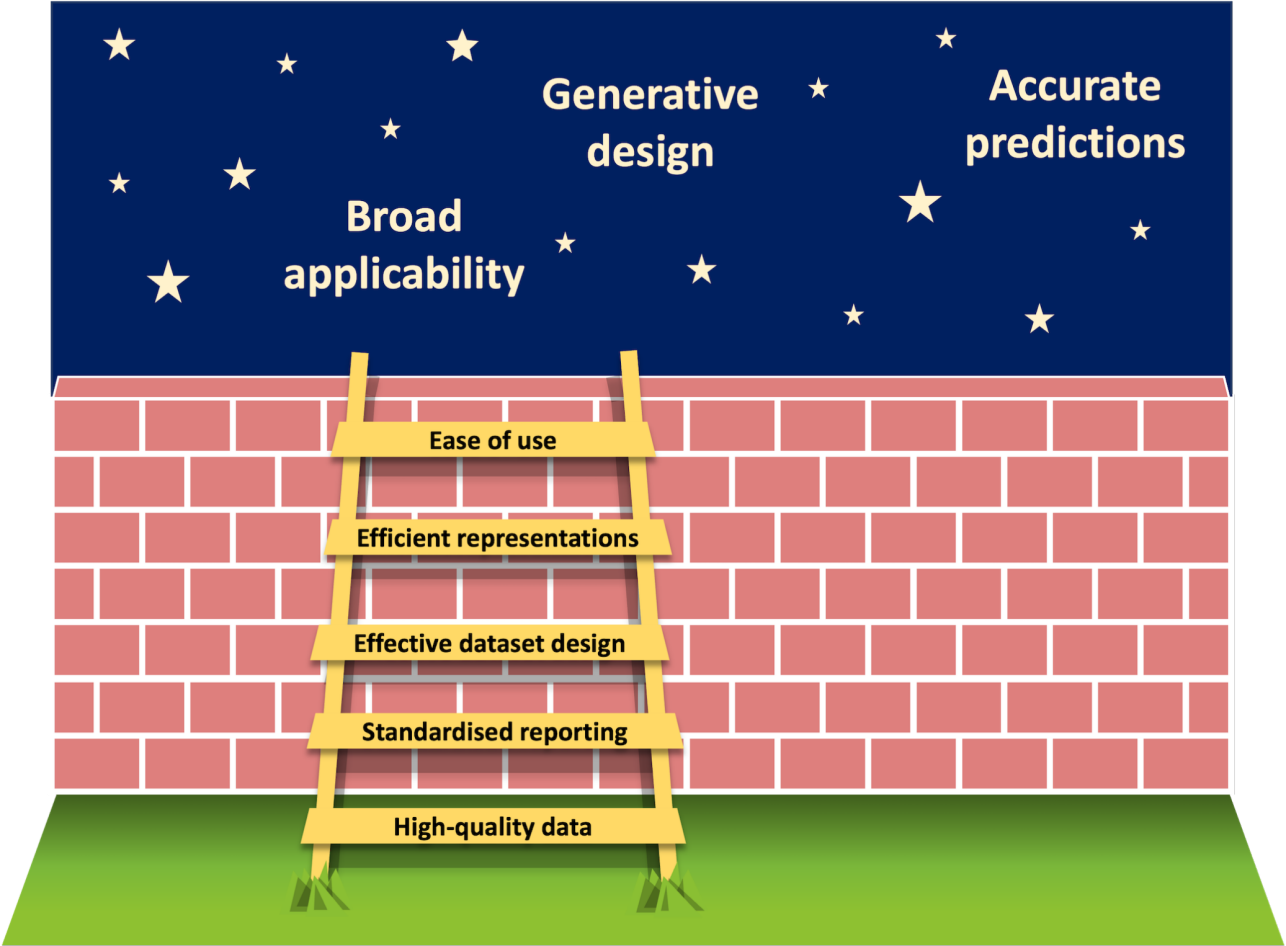
Schematic depiction of required developments in order to overcome current limitations of ML for organocatalysis.

One common bottleneck for further improvements and the wider application of statistical tools is the generation and availability of high-quality data [182] (Figure 18). As the bottlenecks are prevalent throughout the sub-disciplines of homogeneous catalysis, we expect that developments for the application of ML in one area will have a strong influence across the whole domain.

The utilisation of electronic lab notebooks [183–185] and the adoption of standardised formats for collecting and sharing data such as the Open Reaction Database (ORD) scheme could significantly improve the broadness of available data sets [42–43,186–188]. Moreover, standardised protocols for performing experiments, for example for probing the robustness or the sensitivity of a reaction [189–191], as well as the selection of the substrate scope can help to provide valuable information in a reproducible fashion [192–193].

Further, this also requires a paradigm shift towards keeping track of and publishing all conducted experiments, regardless of whether the expected outcome was achieved or not. While HTE campaigns typically yield a broader distribution of reaction outcomes [67], unsuccessful reactivity from traditional "benchtop" chemistry is only rarely reported. Nevertheless, authors are beginning to include a selection of "unsuccessful substrates" in the supporting information [194–198]. In this context, it is necessary to highlight the importance of publishing data in accordance with the FAIR (Findable, Accessible, Interoperable, Reusable) principles to allow for wide usage by the community. Importantly, this does not only apply to experimental work, but also all results from data-driven modelling.

In terms of data set design, Bayesian optimisation bears the potential to maximise the information gained by ML algorithms without the need for extensive experimental effort. In combination with closed-loop high-throughput experimentation, this would allow for fast access to data that cover the problem space adequately and thereby enable optimal modelling. Current challenges for automation pipelines include the purification and analysis of the reaction outcome [199], which is particularly challenging in asymmetric organocatalysis. Due to its relevance for industrial processes however, we expect an increased interest in HTE platforms specifically tailored to organocatalysis, especially (organo-)photocatalysis [200]. In this context, flow chemistry could provide a promising platform to enable closed-loop, multi-objective optimisations and facile scale-up of reactions [201]. With ML tools becoming increasingly accessible for non-experts through easy-to-use interfaces [202–203], their application is expected to gain greater popularity and be integrated into existing routines [204]. This could involve ML-guided catalyst screening, obtaining entries for the substrate scope through unsupervised learning or ML-based reaction condition optimisation. This development will be supported through the advent of LLMs and their incorporation into chemical workflows [176,178] which increase the accessibility of ML tools for synthetic chemistry. While a low entry barrier does not make the knowledge of statistics and coding (primarily in Python) redundant, the abundance of online tutorials and courses on ML allows also non-experts to acquire fundamental skills and to apply such techniques to their own problems. As statistical and coding competencies are becoming more relevant to scientists, courses focused on these fundamentals are being continuously integrated in chemistry curricula at universities.

The last decade has shown the pace at which data-driven tools can be utilised in organocatalysis and led to powerful tools that can augment synthetic chemists. Most works have focused on enantioselectivity as the quantity of interest. Recently, many works have also applied ML for investigating privileged organocatalytic systems. However, there are other objectives that are worth considering when developing a reaction, for example sustainability, complexity, or cost aspects. In this regard future work might involve multi-objective optimisation schemes and generative modelling to account for the plethora of requirements in reaction and process development. Moreover, recent trends in organocatalysis, such as photocatalysis, halogen-bonding, or cooperative catalysis [205], provide new synthetic opportunities, whose advancements are expected to be supported through data-driven modelling.

## Data Availability

Data sharing is not applicable as no new data was generated or analyzed in this study.
